# Clinical characteristics and prognosis of 196 Chinese patients with colon cancer

**DOI:** 10.3389/fsurg.2022.1008149

**Published:** 2023-01-06

**Authors:** Lunjin Yao, Huihui Zhang, Weipeng Wang, Xiaoxia An, Zhiqiang Cheng, Xiang Zhang, Kexin Wang, Binbin Zhang

**Affiliations:** ^1^ Department of General Surgery, Qilu Hospital, Shandong University, Jinan, China; ^2^ Department of Nursing, Qilu Hospital of Shandong University De Zhou Hospital, Dezhou, China

**Keywords:** colon cancer, prognostic factors, cox proportional hazard regression, tumor node metastasis (TNM) staging, tumor makers

## Abstract

**Aims:**

To analyze the clinical characteristics and prognostic factors of Chinese patients with colon cancer.

**Methods:**

A retrospective analysis of the records of patients with colon cancer underwent surgery between 2014 and 2017 was performed. Univariate analysis in combination with Cox proportional hazard regression model was used to analyze the survival data, so as to reveal the prognostic factors of colon cancer. Data record was based on a standard data form. SPSS version 26.0 was used for data analysis (SPSS, Chicago, IL, United States).

**Results:**

The 3-year survival rate and the 5-year survival rate was 79.3% and 68.2%, respectively. Univariate analysis showed that radical surgery, laparoscopic surgery, ascites, swollen lymph nodes at the root of the mesentery, liver metastases, nerve invasion, vascular invasion, tumor node metastasis (TNM) staging, positive level of carbohydrate antigen (CA) 19-9, CA125, CA72-4 and combined detection were positive factors in the prognosis of colon cancer (*P* < 0.05). Multivariate analysis showed that radical surgery and TNM staging were independent factors affecting the prognosis of patients with colon cancer (*P* < 0.05).

**Conclusion:**

Radical surgery and TNM staging have a significant impact on the prognosis of patients with colon cancer.

## Introduction

Globally, colorectal cancer (CRC), a familiar malignant tumor, remains to be one of commonest causes of cancer-related death. In the United States, it is estimated that 151,030 new cases and 52,580 deaths of CRC will occur in 2022 (1). In China, 555,000 new cases of CRC were reported in 2020, accounting for 9.9% of all new malignant tumors, with the mortality rate of 12.0/100,000. CRC has already been the third largest malignant tumor (2), ranking the 5th among the commonest causes of cancer-related death in China (3–5).

In recent years, based on the simultaneous resection of liver metastases and advances in surgical techniques, the 5-year overall survival rate of CRC patients has been improved, and patients with advanced CRC have obtained significant survival benefits (6). Surgery, despite of its role as the first choice for CRC patients, ceases to be the only option to prolong their survival time, with the development of adjuvant therapy. Currently, neoadjuvant therapy, targeted therapy and immunotherapy have greatly enriched the treatment mode of CRC and improved its prognosis. The emergence of further treatment options puts forward higher demands for clinicians. For CRC patients, the accurate prediction of the prognosis is based on the full understanding of the fact that clinical characteristics varied with patients and tumors, clinicians therefore need to formulate the most appropriate therapeutic approach to prolong the survival time as much as possible (7). In addition, different regions and different medical units possess their own advantages in the treatment concepts. MDT (Multi-disciplinary Consultation), carried out by the General Surgery Department of Qilu Hospital of Shandong University, gathers the superior resources of general surgery, oncology, imaging and radiology and other departments, forming a unique CRC treatment system, with a large number of surgeries and regional representation. Therefore, making use of the existing data in Qilu Hospital of Shandong University, we conducted a retrospective analysis of an uncontrolled cohort and investigated the related factors affecting the prognosis of CRC patients.

## Materials and methods

### Patients

From June 2014 to February 2017, a total of 196 patients with colon cancer underwent surgery in the General Surgery Department of Qilu Hospital of Shandong University were included in our database with their informed consent. Thereinto, 8 patients lost follow-up, and 188 patients were finally enrolled in the study.

### Clinical data

All data used in this study have been approved by Qilu Hospital of Shandong University. The clinical data included baseline information(gender, age of diagnosis, family history of CRC, ascites, swollen lymph nodes at the root of the mesentery, liver metastases), surgical approach(radical surgery, laparoscopic surgery), tumor characteristics(tumor location, tumor size, histological type, tumor differentiation, gross type of tumor, nerve invasion, vascular invasion, depth of invasion, number of metastatic lymph nodes, distant metastasis, TNM staging), tumor markers[carcinoembryonic antigen (CEA), CA19-9, CA125, CA72-4 and combined detection positive level] and adjuvant chemotherapy (Table 1). This study included patients with stages I–IV colon cancer. Specimens were fixed with formalin and stained with hematoxylin-eosin (HE) for histopathological evaluation. The 8th editions of the Union for International Cancer Control (UICC) classification were used to categorize colon carcinomas. Right-sided colon cancer (RCC) was defined as tumors located in the cecum, ascending colon, hepatic flexure or transverse colon, and left-sided colon cancer (LCC) was defined as tumors located in the splenic flexure, descending colon or sigmoid colon. The detection method for tumor markers was to take 2 ml of cubital venous blood on an empty stomach in the morning before surgery, separate the serum, and detect the levels of CEA, CA19-9, CA125 and CA72-4 according to standard operating procedures. Reference value range was as follows: CEA:0–5 ng/ml, CA19-9:0–34 U/ml, CA125:0–35 U/ml, CA72-4:0–6.9 U/ml. Positive combined detection was defined as any positive level of CEA, CA 19-9, CA125 and CA72-4.

**Table 1:** Basic data for patients with colon cancer.

Basic data	Frequency (*n*, %)
**Sex**	
Female	76 (40.4)
Male	122 (59.6)
Age^a^ (year)	61.6 ± 12.4
**Family history of CRC**	
Negative	165 (87.7)
Positive	23 (12.3)
**Radical surgery**	
Yes	178 (94.7)
No	10 (5.3)
**Laparoscopic surgery**	
No	46 (24.5)
Yes	142 (75.5)
**Ascites**	
Negative	176 (93.6)
Positive	12 (6.4)
**Swollen lymph nodes at the root of the mesentery**	
Negative	122 (64.9)
Positive	66 (35.1)
**Liver metastases**	
Negative	175 (93.1)
Positive	13 (6.9)
**Tumor location**	
RCC	87 (46.3)
LCC	101 (53.7)
**Tumor size**	
<5 cm	90 (47.9)
≥5 cm	90 (47.9)
Undefined	8 (4.3)
**Histological type**	
Adenocarcinoma	134 (71.3)
Mucinous adenocarcinoma	14 (7.4)
Adenocarcinoma + Mucinous adenocarcinoma	34 (18.1)
Signet ring cell carcinoma	3 (1.6)
Undefined	3 (1.6)
**Tumor differentiation**	
Well	16 (8.5)
Well- Moderate	15 (8.0)
Moderate	118 (62.8)
Moderate-Poor	13 (6.9)
Poor	11 (5.9)
Undefined	15 (8.0)
**Gross type of tumor**	
Ulcer	122 (64.9)
Massive	34 (18.1)
Infiltrate	13 (6.9)
Undefined	19 (10.1)
**Nerve invasion**	
Negative	183 (97.3)
Positive	3 (1.6)
Undefined	2 (1.1)
**Vascular invasion**	
Negative	178 (94.7)
Positive	8 (4.3)
Undefined	2 (1.1)
**Depth of invasion**	
T1	9 (4.8)
T2	11 (5.9)
T3	82 (43.6)
T4	83 (44.1)
Undefined	3 (1.6)
**Numbers of metastatic lymph nodes**	
N0	99 (59.6)
N1a	24 (14.5)
N1b	22 (13.3)
N2a	13 (7.3)
N2b	8 (4.8)
**Distant metastasis**	
M0	141 (75.0)
M1	47 (25.0)
**TNM staging**	
I	13 (6.9)
II	75 (39.9)
III	54 (28.7)
IV	46 (24.5)
**CEA**	
≤5 ng/ml	115 (61.2)
>5 ng/ml	56 (29.8)
Undefined	17 (9.0)
**CA19-9**	
≤34 U/ml	140 (74.5)
>34 U/ml	31 (16.5)
Undefined	17 (9.0)
**CA125**	
≤35 U/ml	151 (80.3)
>35 U/ml	13 (6.9)
Undefined	24 (12.8)
**CA72-4**	
≤6.9 U/ml	119 (63.3)
>6.9 U/ml	37 (19.7)
Undefined	32 (17.0)
**Combined detection**	
Negative	82 (43.6)
Positive	89 (47.3)
Undefined	17 (9.0)
**Adjuvant chemotherapy**	
Positive	99 (52.7)
Negative	31 (16.5)
Undefined	58 (30.9)

RCC, Right-sided colon cancer; LCC, Left-sided colon cancer; TNM, Tumor node metastasis; CEA, Carcinoembryonic antigen; CA, Carbohydrate antigen.

^a^ Data are expressed as mean ± SD.

### Follow-up duration

Except 8 patients lost the follow-up, the remaining 188 patients received regularly postoperative follow-up. The follow-up deadline was set on October 2021 or the time of death. Survival time was defined as the time from admission to death or the end of follow-up. All patients were followed up at 3-month intervals for the first 2 years, and 6-month intervals for 3–5 years. The median follow-up period was 60.81 months.

### Statistical analysis

All data were recorded using standard data form and analyzed using SPSS version 26.0 (SPSS, Chicago, IL, United States). The survival curve was calculated by Kaplan-Meier method. Log rank test was used to assess differences in survival. Cox proportional risk analysis was performed to identify significant and independent predictors of univariate risk ratios and disease-specific survival. Spearman rank correlation test was used for correlation analysis. Stepwise procedure was set to a threshold of 0.05. *P* values are derived from two-tailed tests. *P* < 0.05 was considered statistically significant.

## Results

Of 188 patients with colon cancer, the 3-year and 5-year overall survival rate were 79.3% and 68.2%, respectively, with the mean overall survival of 1534.13 ± 552.94 days. 76 were female and 122 were male, and the gender difference in survival time was not statistically significant (*P* = 0.69).

Patients in this study were aged 15–86 years, with an average age of 61.6 ± 12.4 years. Patients were divided into four groups according to age of diagnosis: age1: ≤35 years, age2: 36–59 years, age3: 60–74 years, and age4: ≥75 years. The 3-year survival rates of the four groups of patients were 75.0%, 76.8%, 83.3%, and 76.3%, respectively, while the 5-year survival rates were 75.0%, 72.5%, 69.4%, and 56.9%, respectively, the age difference in survival time was not statistically significant (*P* = 0.476).

In this study, a total of 23 patients had a family history of CRC, but their survival time was not significantly different from that of the group without family history of CRC. The 3-year survival rates of patients with and without family history were 79.4% and 78.3%, respectively. The 5-year survival rates were 68.4% and 67.8%, respectively (*P* = 0.936).

There were 87 RCC patients and 101 LCC patients enrolled in our study. The results showed that the 3-year and 5-year survival rate of RCC patients were 78.2% and 66.3%, respectively. The 3-year and 5-year survival rate of LCC patients were 80.2% and 69.7%, respectively. The results were not statistically significant (*P* = 0.414).

**Figure 1:**
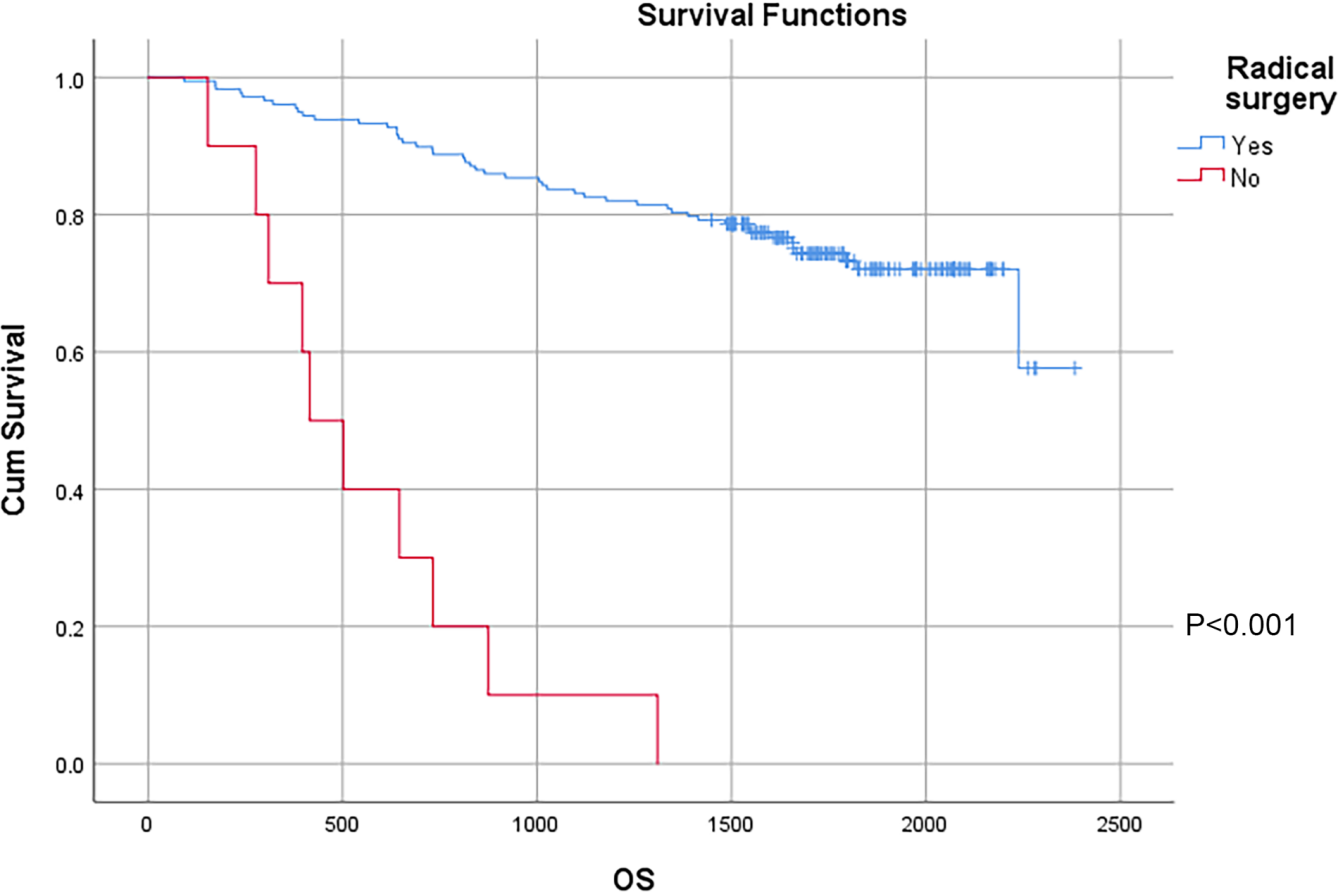
Survival curves of colon cancer patients in different radical surgery groups.

**Figure 2:**
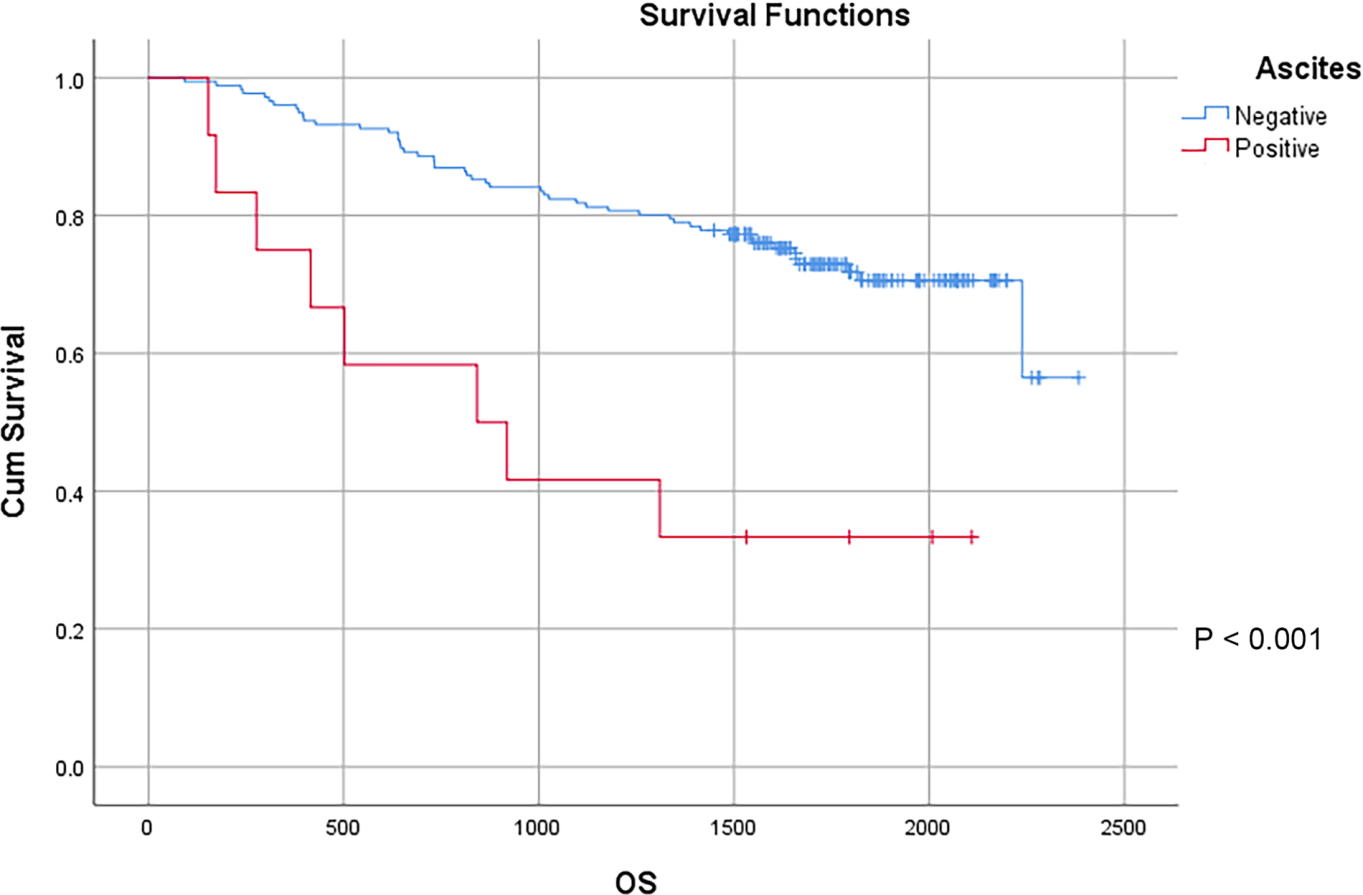
Survival curves of colon cancer patients in different ascites groups.

**Figure 3:**
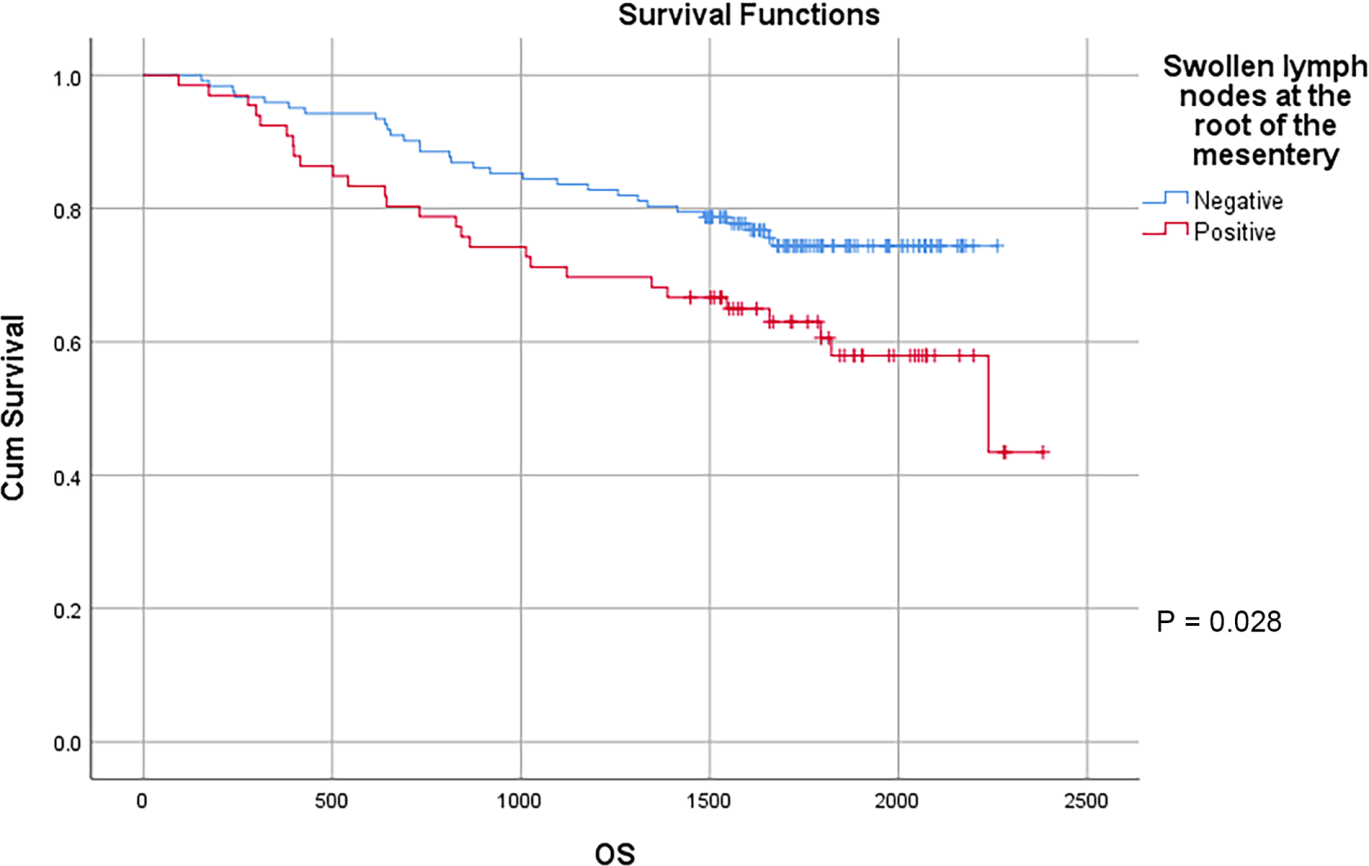
Survival curves of colon cancer patients in different swollen lymph nodes at the root of the mesentery groups.

**Figure 4:**
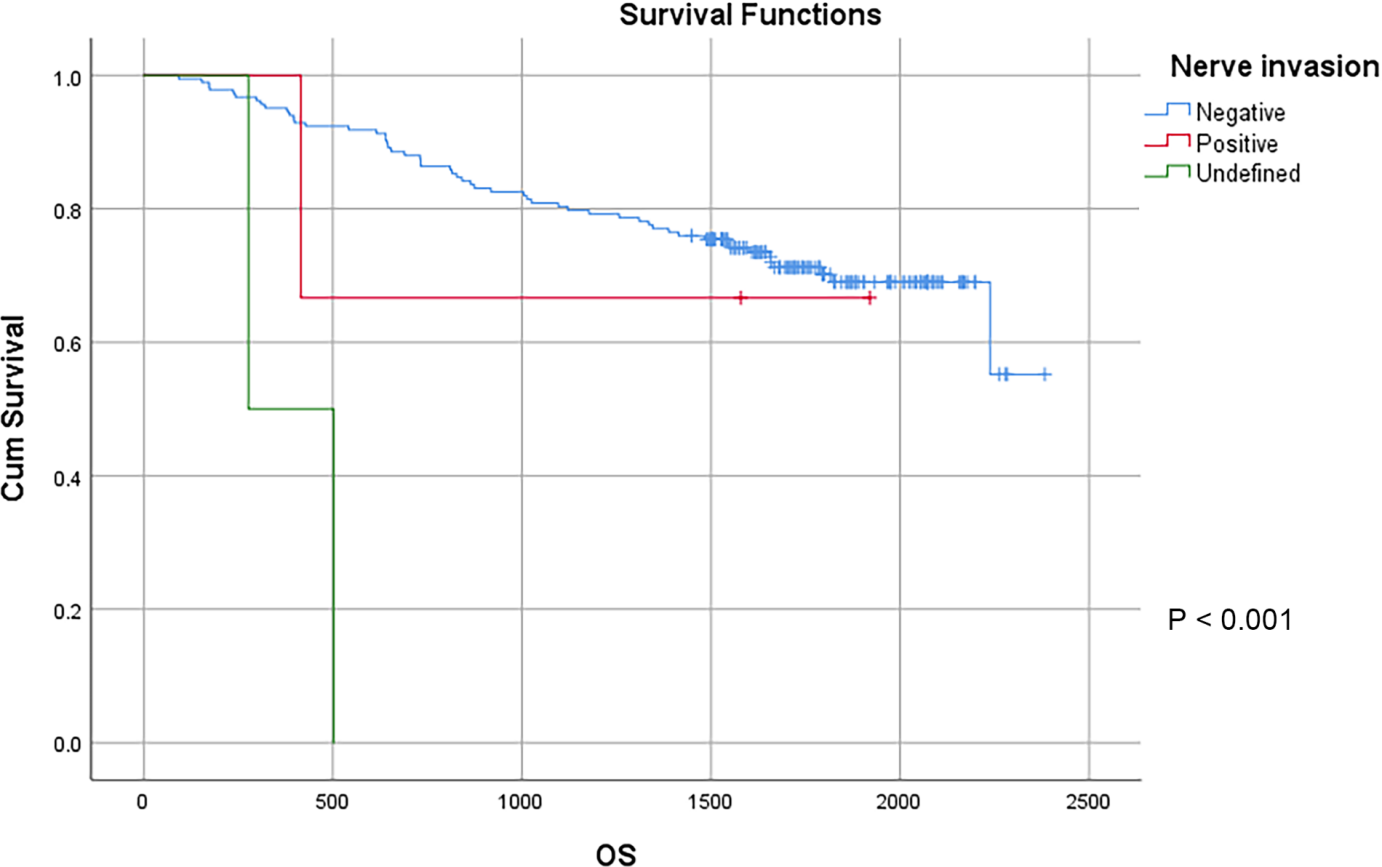
Survival curves of colon cancer patients in different nerve invasion groups.

**Figure 5:**
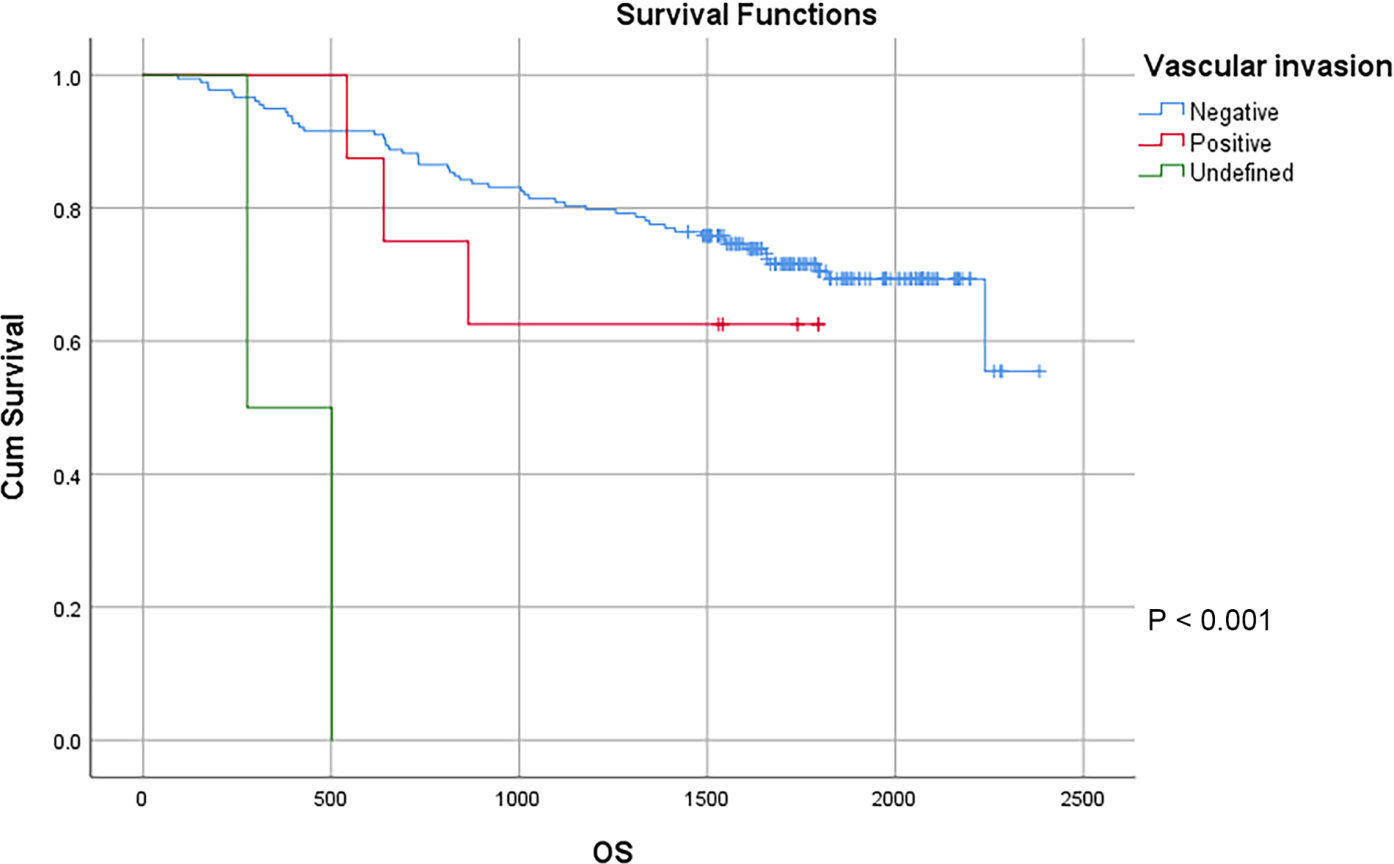
Survival curves of colon cancer patients in different vascular invasion groups.

In this study, a total of 178 patients underwent radical surgery, and 10 patients underwent palliative surgery or exploratory surgery because of advanced tumor or serious complications. The results showed that the postoperative survival time of palliative surgery or exploratory surgery group was significantly lower than that of radical surgery group. The 3-year and the 5-year survival rate of the radical surgery group were 83.1% and 72.1%, respectively. The 3-year and the 5-year survival rate of palliative surgery group or exploratory surgery group were 10.0% and 0%, respectively. From the above result, the difference of the 3-year and the 5-year survival rate between the two groups was not statistically significant (*P* < 0.001) (Figure 1). Our study demonstrated the radical surgery to be one of the key factors affecting the prognosis of patients with colon cancer. Due to the advantages of less trauma and rapid postoperative recovery, laparoscopic-assisted radical resection of colon cancer has currently become a routine operation in Qilu Hospital of Shandong University. In our study, a total of 142 patients underwent laparoscopic surgery, with the 3-year and the 5-year survival rate of 83.1% and 74.4%, respectively. As a comparison, a total of 46 patients underwent traditional laparotomy, with a 3-year and 5-year survival rate of 67.4% and of 50.4%, respectively. On the basis of the advanced tumor staging, which would increase the difficulty of laparoscopic surgery, and awful general condition, as well as the underlying diseases and unbearable laparoscopic surgery, the patients had to receive traditional laparotomy rather than laparoscopic surgery. Therefore, compared with the patients underwent laparoscopic surgery, patients underwent traditional laparotomy exhibited a worse prognosis (*P* < 0.01). In addition, ascites, swollen lymph nodes at the root of the mesentery, liver metastases, nerve invasion, vascular invasion were considered as potential factors influencing the prognosis of patients with colon cancer (*P* < 0.05) (Table 2) (Figures 2–5).

**Table 2:** Univariate analysis of the prognostic factors for patients with colon cancer.

Univariate analysis				
	*n*	3-YSR	5-YSR	*P* value
Sex				**0** **.** **069**
Female	76	72.4%	61.3%	
Male	122	83.9%	72.9%	
Age				**0** **.** **476**
Age1 (≤35)	4	75.0%	75.0%	
Age2 (36–59)	69	76.8%	72.5%	
Age3 (60–74)	84	83.3%	69.4%	
Age4 (≥75)	31	74.2%	56.9%	
Family history of CRC				**0** **.** **936**
Negative	165	79.4%	68.4%	
Positive	23	78.3%	67.8%	
Radical surgery				**<0** **.** **001**
Yes	178	83.1%	72.1%	
No	10	10.0%	10.0%	
Laparoscopic surgery				**0** **.** **002**
No	46	67.4%	50.4%	
Yes	142	83.1%	74.4%	
Ascites				**<0** **.** **001**
Negative	176	81.8%	70.6%	
Positive	12	41.7%	33.3%	
Swollen lymph nodes at the root of the mesentery				**0** **.** **028**
Negative	122	83.6%	74.4%	
Positive	66	71.2%	57.9%	
Liver metastases				**<0** **.** **001**
Negative	175	83.4%	72.2%	
Positive	13	23.1%	15.4%	
Tumor site				**0** **.** **414**
RCC	87	78.2%	66.3%	
LCC	101	80.2%	69.7%	
Tumor size				**0** **.** **676**
<5 cm	90	80.0%	68.7%	
≥5 cm	90	80.0%	68.4%	
Undefined	8	62.5%	62.5%	
Histological type				**0** **.** **330**
Adenocarcinoma	134	82.1%	68.1%	
Mucinous adenocarcinoma	14	78.6%	643%	
Adenocarcinoma + mucinous adenocarcinoma	34	73.5%	73.5%	
Signet ring cell carcinoma	3	66.7%	66.7%	
Undefined	3	33.3%	33.3%	
Tumor differentiation				**0** **.** **120**
Well	16	81.3%	75.0%	
Well- moderate	15	100%	93.3%	
Moderate	118	81.4%	67.0%	
Moderate-*p*oor	13	53.8%	53.8%	
Poor	11	72.7%	72.7%	
Undefined	15	66.7%	53.3%	
Gross type of tumor				**0** **.** **501**
Ulcer	122	79.5%	68.5%	
Massive	34	79.4%	62.7%	
Infiltrate	13	69.2%	57.7%	
Undefined	19	84.2%	84.2%	
Nerve invasion				**<0** **.** **001**
Negative	183	80.3%	69.0%	
Positive	3	66.7%	66.7%	
Undefined	2	0%	0%	
Vascular invasion				**<0** **.** **001**
Negative	178	80.9%	69.3%	
Positive	8	62.5%	62.5%	
Undefined	2	0%	0%	
Adjuvant chemotherapy				**0** **.** **004**
Positive	99	79.8%	68.7%	
Negative	31	93.5%	90.2%	
Undefined	58	70.7%	47.9%	

3-YSR, 3-year accumulative survival rate; 5-YSR, 5-year accumulative survival rate; CRC, Colorectal cancer; RCC, Right-sided colon cancer; LCC, Left-sided colon cancer.

**Figure 6:**
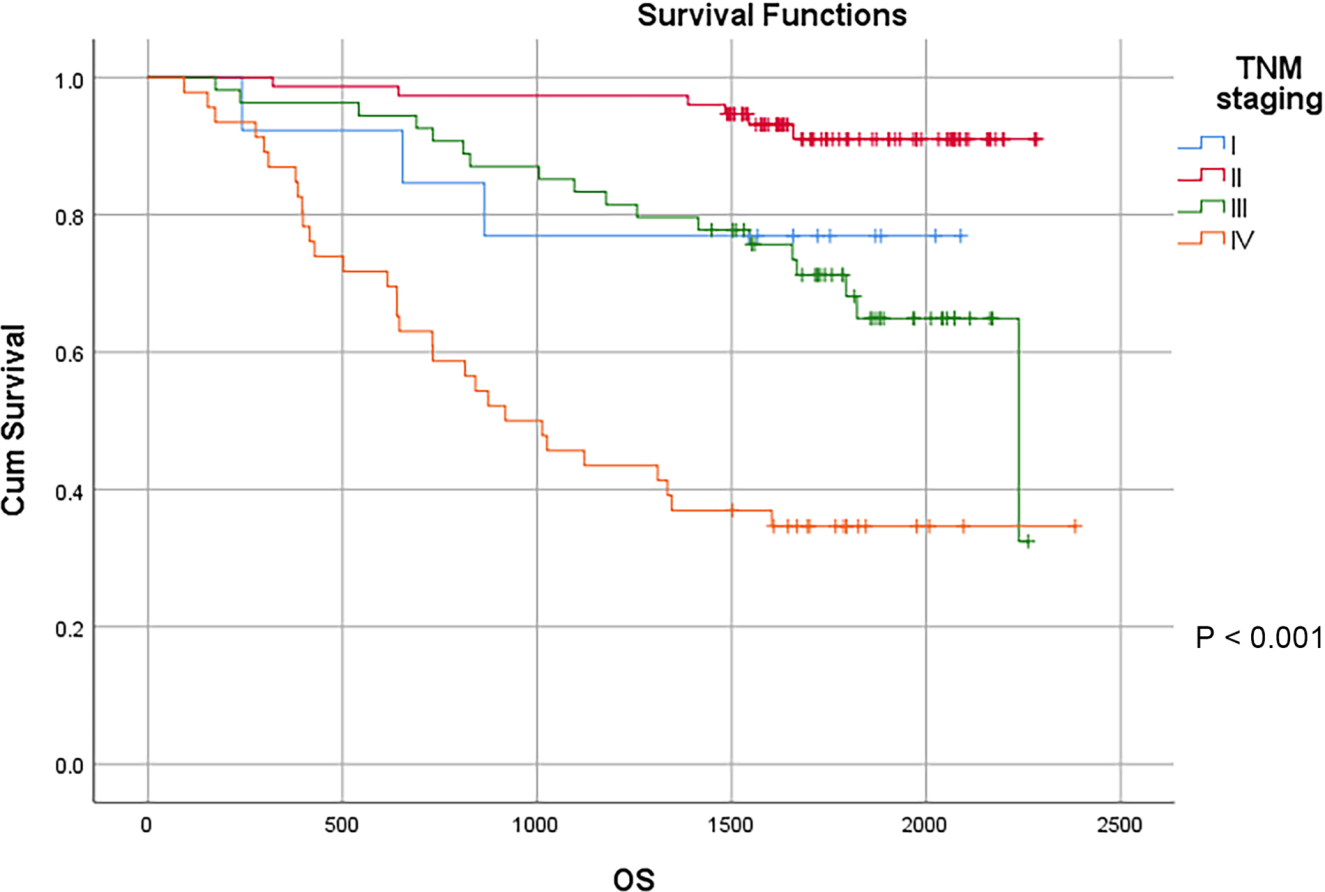
Survival curves of colon cancer patients in different TNM staging groups.

TNM staging is a globally recognized tumor staging standard, and the main representative of tumor staging in China as well. The results showed that the depth of primary tumor invasion remarkably affected the prognosis of patients with colon cancer. The 3-year survival rates of T1, T2, T3 and T4 patients were 88.9%, 72.7%, 87.8% and 73.5%, respectively, and the 5-year survival rate of T1, T2, T3 and T4 patients were 88.9%, 72.7%, 71.8% and 62.8%, respectively, with statistically significant difference (*P* < 0.01). With the increase of the estimated number of positive lymph nodes and distant metastases, the survival of patients with colon cancer gradually declined. According to the 2021 CSCO Guidelines for Colorectal Cancer, the number of lymph nodes to be dissected should be ≥12. In our study, 166 cases remained after removing less than 12 lymph nodes. In the remaining samples, the 3-year survival rate of N0, N1a, N1b, N2a and N2b patients were 93.9%, 87.5%, 68.2%, 53.8% and 37.5%, respectively, and the 5-year survival rate of N0, N1a, N1b, N2a and N2b patients were 84.9%, 67.5%, 42.0%, 46.2% and 37.5%, respectively, with statistically significant difference (*P* < 0.01). The 3-year survival rate of M0 and M1 were 90.1% and 46.8%, respectively, and the 5-year survival rate of M0 and M1 were 79.1% and 35.9%, respectively, with statistically significant difference (*P* < 0.01). The results showed the significant impact of TNM staging on the prognosis of patients. The 3-year survival rate of stage I, II, III and IV patients were 76.9%,97.3%,83.3% and 45.7%, respectively, and the 5-year survival rate of stage I, II, III and IV patients were 76.9%, 91.0%, 64.9% and 34.6%, respectively, with statistically significant difference (*P* < 0.01) (Table 3) (Figure 6).

**Table 3:** Univariate analysis of TNM staging for tumor from patients with colon cancer.

TNM staging				
	*n*	3-YSR	5-YSR	*P* value
Depth of invasion				**<0** **.** **001**
T1	9	88.9%	88.9%	
T2	11	72.7%	72.7%	
T3	82	87.8%	71.8%	
T4	83	73.5%	62.8%	
Undefined	3	0.00%	0.00%	
Numbers of metastatic lymph nodes				**<0** **.** **001**
N0	99	93.9%	84.9%	
N1a	24	87.5%	67.5%	
N1b	22	68.2%	42.0%	
N2a	13	53.8%	46.2%	
N2b	8	37.5%	37.5%	
Distant metastasis				**<0** **.** **001**
M0	141	90.1%	79.1%	
M1	47	46.8%	35.9%	
TNM staging				**<0** **.** **001**
I	13	76.9%	76.9%	
II	75	97.3%	91.0%	
III	54	83.3%	64.9%	
IV	46	45.7%	34.6%	

3-YSR, 3-year accumulative survival rate; 5-YSR, 5-year accumulative survival rate; TNM, Tumor node metastasis.

**Figure 7:**
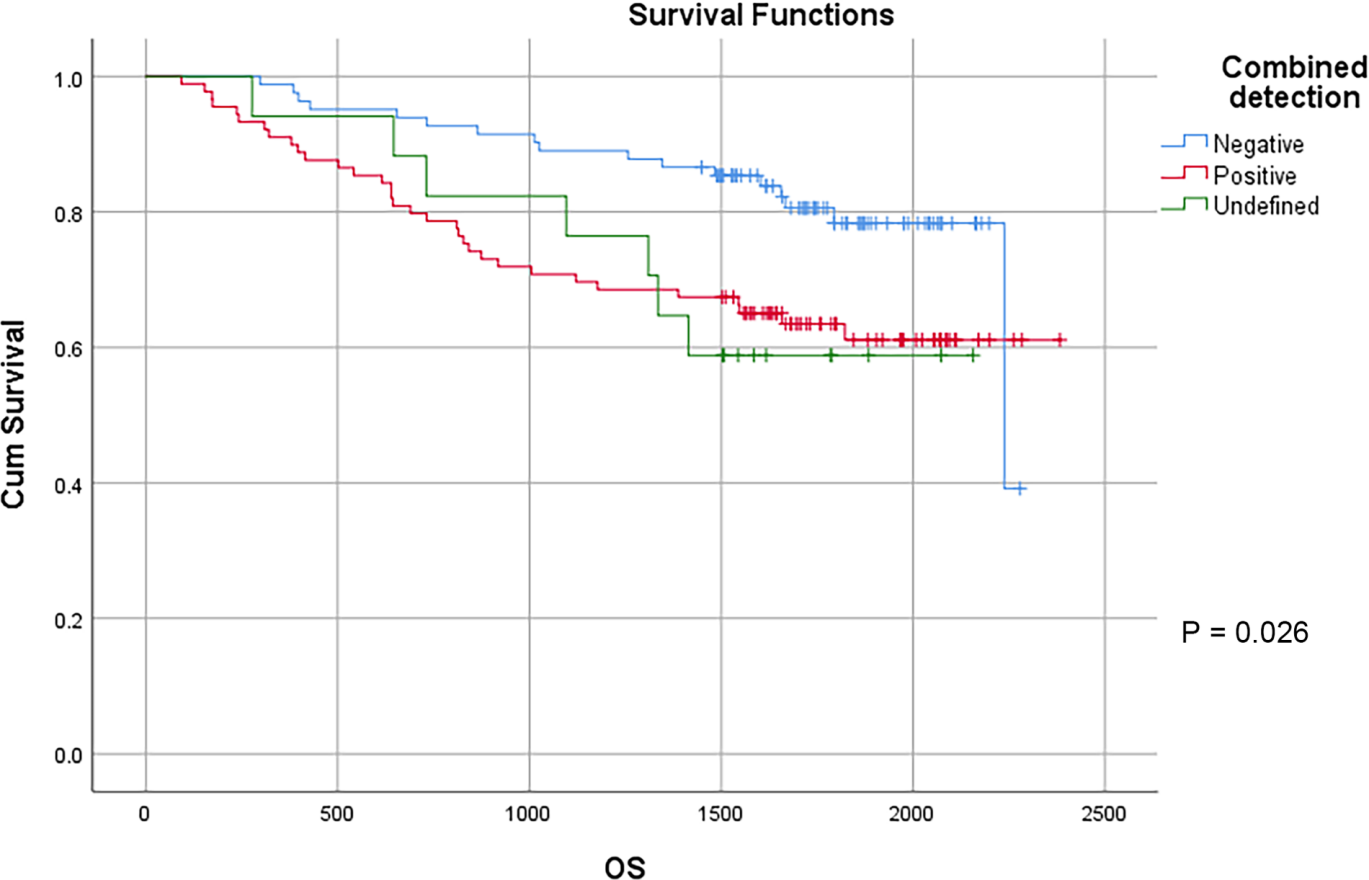
Survival curves of colon cancer patients in different combined detection groups.

CEA, CA 19-9, CA125 and CA72-4 are common gastrointestinal tumor markers. Our study showed that preoperative CEA expression level did not affect the survival of patients with colon cancer. The 3-year survival rates of patients with preoperative CEA ≤ 5 ng/ml and CEA > 5 ng/ml were 82.6% and 73.2%, respectively, and the 5-year survival rates of patients with preoperative CEA ≤ 5 ng/ml and CEA > 5 ng/ml were 74.9% and 58.1%, respectively (*P* = 0.88). However, preoperative CA19-9, CA125, CA72-4 exhibited a certain effect on the survival of patients with colon cancer. The 3-year survival rates of patients with preoperative CA19-9 ≤ 34 U/ml and CA19-9 > 34 U/ml were 83.6% and 61.3%, respectively, and the 5-year survival rates of patients with preoperative CA19-9 ≤ 34 U/ml and CA19-9 > 34 U/ml were 73.9% and 48.0% (*P* < 0.01), respectively. The 3-year survival rates of patients with preoperative CA125 ≤ 35 U/ml and CA125 > 35 U/ml were 83.4% and 38.5%, respectively, and the 5-year survival rates of patients with preoperative CA125 ≤ 35 U/ml and CA125 > 35 U/ml were 71.9% and 38.5%, respectively, with statistically significant difference (*P* < 0.01). The 3-year survival rates of patients with preoperative CA72-4 ≤ 6.9 U/ml and CA72-4 > 6.9 U/ml were 85.7% and 62.2%, respectively, and the 5-year survival rate of patients with preoperative CA72-4 ≤ 6.9 U/ml and CA72-4 > 6.9 U/ml were 73.6% and 54.6%, respectively, with statistically significant difference (*P* = 0.040). Our study showed that the 3-year survival rates of patients with negative and positive combined detection were 89.0% and 70.8%, respectively, and the 5-year survival rates of patients with negative and positive combined detection were 78.3% and 61.1%, respectively, with statistically significant difference (*P* = 0.026) (Table 4) (Figure 7).

**Table 4:** Univariate analysis of tumor makers for patients with colon cancer.

Tumor Markers				
	*n*	3-YSR	5-YSR	*P* value
CEA				**0** **.** **088**
≤5 ng/ml	115	82.6%	74.9%	
>5 ng/ml	56	73.2%	58.1%	
Undefined	17	76.5%	58.8%	
CA19-9				**0** **.** **007**
≤ 34 U/ml	140	83.6%	73.9%	
>34 U/ml	31	61.3%	48.0%	
Undefined	17	76.5%	58.8%	
CA125				**<0** **.** **001**
≤35 U/ml	151	83.4%	71.9%	
>35 U/ml	13	38.5%	38.5%	
Undefined	24	75.0%	62.5%	
CA72-4				**0** **.** **040**
≤6.9 U/ml	119	85.7%	73.6%	
>6.9 U/ml	37	62.2%	54.6%	
Undefined	32	75.0%	65.6%	
Combined detection				**0** **.** **026**
Negative	82	89.0%	78.3%	
Positive	89	70.8%	61.1%	
Undefined	17	76.5%	58.8%	

3-YSR, 3-year accumulative survival rate; 5-YSR, 5-year accumulative survival rate; CEA, Carcinoembryonic antigen; CA: Carbohydrate antigen.

We further calculated the relationship between the expression levels of CEA, CA125, CA72-4 and CA19-9 and factors such as age, tumor differentiation, tumor size, TNM staging and lymph node metastasis. The results showed that the expressions of CEA, CA125, CA72-4 and CA19-9 were independent of age (*P* > 0.05). The lower the degree of tumor differentiation, the higher the expression level of CA125 (*P* = 0.008), while the expression levels of CEA, CE72-4 and CA19-9 were not related to the degree of tumor differentiation (*P* > 0.05). The larger the tumor size, the higher the expression level of CA72-4 (*P* = 0.019), while the expressions of CEA, CA125 and CA19-9 were not related to the tumor size (*P* > 0.05). The later the TNM staging, the higher the expression levels of CEA, CA125, CA72-4 and CA19-9 (*P* < 0.05). The higher the number of lymph node metastasis, the higher the expression levels of CEA, CA125 and CA19-9 (*P* < 0.05), but the expression level of CA 72-4 was not related to the number of lymph node metastasis (*P* > 0.05) (Table 5).

**Table 5:** Spearman rank correlation test for tumor makers of patients with colon cancer.

	CEA		CA19-9		CA125		CA72-4	
	r_s_	*P*	r_s_	*P*	r_s_	*P*	r_s_	*P*
Age	0.092	0.228	0.111	0.145	0.077	0.320	−0.115	0.147
Differentiation	0.045	0.575	0.145	0.068	0.213	0.008	0.068	0.413
Tumor size	0.070	0.369	0.019	0.809	−0.026	0.747	0.180	0.026
TNM staging	0.200	0.008	0.207	0.006	0.289	0.001	0.172	0.029
Lymph node metastasis	0.182	0.023	0.162	0.045	0.210	0.010	0.159	0.058

TNM, Tumor node metastasis; CEA, Carcinoembryonic antigen; CA, Carbohydrate antigen.

We also counted the positive rate of CEA, CA125, CA72-4 and CA199 levels and the positive rate of combined detection. The results showed that the positive rates of CEA, CA19-9, CA125 and CA72-4 in 188 patients were 32.7%, 18.1%, 23.9% and 23.7%, respectively. The positive rate of combined detection was 52.0%, which was significantly higher than that of individual indicators, and the difference was statistically significant (*P* < 0.05).

Due to incomplete follow-up data and other reasons, a total of 130 patients knew definitively whether to receive adjuvant chemotherapy. There were 99 patients with adjuvant chemotherapy, with the 3-year and the 5-year survival rate of 79.8% and 68.7%, respectively. 31 patients did not receive adjuvant chemotherapy, with the 3-year and the 5-year survival rate of 93.5% and 90.2%, respectively, the difference was statistically significant (*P* = 0.004).

The survival time of patients receiving adjuvant chemotherapy was shorter than that of patients not receiving adjuvant chemotherapy, since the TNM staging remained later in patients receiving adjuvant chemotherapy was and earlier in patients not receiving adjuvant chemotherapy.

According to our study results, in the overall sample, stage III patients exhibited a shorter survival time than stage II patients. However, among the 99 patients with the definite adjuvant chemotherapy, the 3-year survival rates of stage II and stage III patients were 97.1% and 91.9%, respectively, and the 5-year survival rates of stage II and stage III patients were 89.8% and 75.4%, respectively, with no statistically significant difference (*P* = 0.174). The results showed that adjuvant chemotherapy extended survival in stage III patients to a level similar to that in stage II patients.

We calculated the positive factors influencing the prognosis of patients with colon cancer by univariate analysis, afterwards, we investigated the most significant prognostic factors through multivariate analysis (Cox proportional hazard model).

Radical surgery, ascites, swollen lymph nodes at the root of the mesentery, nerve and vascular invasion, TNM staging, and positive combined detection into COX analysis were included in the study. After removing undefined data, a total of 170 patients remained.

The results showed that radical surgery and TNM staging were independent prognostic factors affecting the prognosis of patients with colon cancer (*P* < 0.05) (Table 6).

**Table 6:** Multivariate analysis (Cox proportional hazard model) of prognostic factors.

Parameter	*P* value	HR	95%CI
Radical surgery	<0.001	8.377	3.011–23.303
Ascites	0.386	1.610	0.548–4.729
Swollen lymph nodes at the root of the mesentery	0.099	1.623	0.913–2.886
Nerve invasion	0.516	0.461	0.045–4.774
Vascular invasion	0.592	1.397	0.412–4.733
TNM staging	0.001	3.779	1.787–7.990
Positive combined detection of tumor makers	0.245	1.459	0.772–2.756

TNM, Tumor node metastasis.

## Discussion

CRC, the third in common cancer in China, is inferior to lung cancer and gastric cancer (2). In addition, CRC remains to be the second leading cause of cancer-related death globally, with 1,931,590 new cases and 935,173 deaths worldwide in 2020 (8). Recent years have witnessed the advancement of surgical techniques and various adjuvant treatments. However, despite of the greatly improved prognosis of CRC patients, the 5-year overall survival rate is still approximately 60%. In our study, the 3-year and the 5-year survival rate of colon cancer patients were 79.3% and 68.2%, respectively.

Compared to traditional laparotomy, laparoscopic surgery can effectively reduce surgical trauma. Laparoscopic surgery restricts the extent of abdominal incisions, avoids manual traction and manipulation of abdominal tissue, and prevents undue blood loss, thus diminishing immune activation and catabolism as a response to surgery (9, 10). The COlon cancer Laparoscopic or Open Resection Study Group (COLOR) has compared laparoscopic and open surgery in patients with colon cancer and showed that laparoscopic surgery had less blood loss, earlier recovery of bowel function, fewer analgesics, and shorter hospital stays than traditional laparotomy (11). Our study has similar results (date not shown). In terms of long-term outcome, there was no significant difference in 3-year disease-free survival and overall survival between patients undergoing laparoscopic surgery and traditional laparotomy (12). However, in our study, patients undergoing traditional laparotomy had a shorter survival time than those undergoing laparoscopic surgery (*P* < 0.01).This may be due to the fact that the COLOR trials is prospectively designed in which patients were randomized to undergo laparoscopic surgery or traditional laparotomy. However, in our study, surgeons preferred to choose traditional laparotomy for patients with advanced tumor staging.

Currently, there remains a controversial issue of the difference in prognosis between RCC and LCC (13). A slew of studies showed lower survival rate in RCC patients than LCC patients (14). According to American Joint Committee on Cancer, RCC patients possessed a statistically significant advantage in relative survival rate compared with LCC patients (15). Benedix et al.'s study showed significant differences in the epidemiological characteristics and histological parameters of RCC and LCC, as well as a worse prognosis of RCC patients than LCC patients (16). According to a study by Wang Hui et al., the 5-year survival rate was 62.58% in 243 RCC patients and 69.41% in 339 LCC patients (17). However, our results showed no significant difference in the prognosis between RCC patients and LCC patients (*P* = 0.414).

Liver metastasis is the most common distant metastasis of CRC, of which the effective treatment is surgical resection. Choti et al.' s study showed that the 5-year survival rate of patients underwent liver metastasis resection was close to 50% (18). However, only 20% to 30% of metastatic CRC patients have metastases confined to the liver (19). For patients with metastatic CRC who cannot receive simultaneous resection of liver metastases at the initial stage, we can make simultaneous resection of liver metastases possible through effective chemotherapy and other effective adjuvant therapies, namely, the conversion therapy. Maeda et al.' s study showed that the survival rate of patients with liver metastasis resection after conversion therapy was similar to that of patients with simultaneous resection of liver metastases (20). When there is rapid breakthrough in molecular biology technology, targeted therapy increasingly features in CRC treatment. Saltz et al.' s study showed that chemotherapy combined with targeted agents such as cetuximab or bevacizumab could significantly prolong the survival time of patients with advanced CRC (21). Research on immunotherapy for CRC has also developed rapidly in recent years. CRC can be divided into two major categories–mismatch repair deficient microsatellite high instability (dMMR-MSI-H)(15%) and mismatch repair proficient microsatellite low instability (pMMR-MSI-L)(85%). In terms of the particularity of dMMR-MSI-H CRC, Sargent et al.' study showed that CRC patients with dMMR-MSI-H did not benefit from adjuvant chemotherapy with single-agent fluorouracils (22). However, due to the high tumor mutation accumulation and immune cell infiltration, dMMR-MSI-H CRC can benefit from PD1 inhibitor immunotherapy. FDA has approved the use of the anti-PD1 antibodies paclizumab and nivolumab for the treatment of dMMR-MSI-H CRC (23).

TNM system is considered to be the current international standard for CRC staging. Our study showed that the significantly decreased depth of infiltration with the increase of the depth of invasion, the number of lymph node metastases and the status of distant metastases, the survival time of patients with colon cancer (*P* < 0.05). Regardless of univariate analysis or COX analysis, TNM staging remains to be an important factor affecting the prognosis of patients with colon cancer. The later the TNM staging, the shorter the survival time of patients (*P* < 0.001).

The study of Zhu et al. showed that the combined detection of CEA, CA19-9, CA72-4 and CA242 can improve the positive detection rate of gastric cancer, which is of great significance in its evaluation of the prognosis (24). In CRC patients, Zhou et al.' s study showed that the combined detection of CEA, CA199 and CA242 exhibited clinical significance for the preoperative diagnosis of CRC, and dynamic detection also has momentous value for judging the treatment effect and prognosis (25). CEA, CA19-9, CA125 and CA72-4 are commonly used tumor markers in Qilu Hospital of Shandong University. In this study, the combined detection of CEA, CA19-9, CA125 and CA72-4 was found to improve the positive detection rate of colon cancer, and the survival time of patients with positive results was significantly lower than that of patients with negative results. Therefore, in the aspect of colon cancer, the combined detection of CEA, CA19-9, CA125 and CA72-4 can not only make up for the shortcomings of different sensitivity and specificity of a single tumor marker, but also facilitate the early diagnosis and treatment, which contributes to taking corresponding treatment measures more timely and prolonging the survival time of patients with colon cancer to a possible extent.

Surgery is the primary choice for the treatment of directly resected CRC. For CRC patients with lymph node metastasis or distant organ metastasis (lung or liver), postoperative adjuvant chemotherapy can improve the prognosis and prolong the survival time to a certain extent. de Gramont et al.' study showed that for patients with metastatic CRC, postoperative adjuvant chemotherapy can more than double the disease-free survival time (26), which was consistent with our findings.

Through COX analysis, radical surgery and TNM staging were confirmed as independent factors affecting the prognosis of patients with colon cancer. Through univariate analysis, other important factors that were potentially found to affect the prognosis of colon cancer patients, including laparoscopic surgery, ascites, swollen lymph nodes at the root of the mesentery, liver metastases, nerve invasion, vascular invasion, TNM staging, positive expression of tumor markers CA19-9, CA125, CA72-4, and positive combined detection of CEA, CA19-9, CA125, and CA72-4 (*P* < 0.05).

This study was limited for the retrospective design and relatively small sample size, which may have limited the generalization of the results.

## Conclusion

With application of neoadjuvant and adjuvant chemo- or radiotherapy as well as laparoscopic technique, the 3-year survival rate and the 5-year survival rate of colon cancer has reached 79.3% and 68.2%, respectively, with radical surgery and TNM staging being major prognostic factors.

## Data Availability

The raw data supporting the conclusions of this article will be made available by the authors, without undue reservation.
